# Distinct NK Cell Signatures Define Prognosis in HPV-Positive Versus HPV-Negative Head and Neck Cancer

**DOI:** 10.3390/cancers18050845

**Published:** 2026-03-05

**Authors:** Rui Li, Fangjia Tong, Huan Liu, Zengchen Liu, Wanlin Li, Yingdong Zhang, Yiman Peng, Shuang Pan, Lanlan Wei, Ning Li, Ming Chu

**Affiliations:** 1 Department of Neurosurgery, Shenzhen Third People’s Hospital, The Second Hospital Affiliated to Southern University of Science and Technology, Shenzhen 518112, China; 2 National Clinical Research Center for Infectious Diseases, Institute for Hepatology, The Third People’s Hospital of Shenzhen, The Second Hospital Affiliated to Southern University of Science and Technology, Shenzhen 518112, China; 3 The First Affiliated Hospital of Harbin Medical University and Department of Endodontics, School of Stomatology, Harbin Medical University, Harbin 150001, China; 4 Biotherapy Clinical Research Center, Shenzhen Third People’s Hospital, Second Hospital Affiliated to Southern University of Science and Technology, Shenzhen 518112, China

**Keywords:** human papillomavirus, head and neck cancer, natural killer cells, tumor microenvironment, immunotherapy

## Abstract

**Background/Objectives:** HPV status is a key prognostic determinant in head and neck squamous cell carcinoma (HNSCC), yet the immunological mechanisms underlying the survival advantage of HPV-positive (HPV^+^) over HPV-negative (HPV^−^) disease remain poorly defined. This study aimed to characterize the tumor-infiltrating natural killer (NK) cell landscape in HPV-stratified HNSCC and identify novel therapeutic targets. **Methods:** We performed an NK-cell-centric re-analysis of published scRNA-seq data from 28 HNSCC patients (10 HPV^+^, 18 HPV^−^; GEO: GSE139324, GSE164690), encompassing NK subset identification, pseudotime trajectory inference, and cell–cell interaction analysis. Key findings were validated by immunohistochemistry (IHC) in an independent cohort of 10 FFPE tissue sections, and prognostic associations were assessed using TCGA-HNSC data. **Results:** Four transcriptionally distinct NK cell subsets were identified: adaptive, cell-killing, CD56^bright^, and virus-responsive. A cytotoxic CX3CR1^+^KLRB1^dim^ NK subset was specifically enriched in HPV^+^ tumors and independently associated with favorable survival. Conversely, HPV^−^ tumors upregulated CLEC2C and CLEC2D ligands on tumor cell surfaces, engaging the inhibitory receptor KLRB1 on NK cells; this CLEC2–KLRB1 axis correlated with suppressed NK activity and poorer prognosis, and was confirmed at the protein level by IHC. **Conclusions:** NK cell function in HNSCC is dichotomously regulated by HPV status. The CX3CR1^+^KLRB1^dim^ subset represents a candidate prognostic biomarker in HPV^+^ disease, and the CLEC2–KLRB1 axis is a targetable immune evasion mechanism in HPV^−^ HNSCC. These insights support the development of HPV-stratified immunotherapies; however, clinical translation requires validation in large, prospectively designed, subsite-matched cohorts to disentangle HPV-specific effects from anatomical site-dependent immune contextures.

## Simple Summary

Head and neck cancer can be caused by human papillomavirus (HPV) infection or by factors such as smoking. Surprisingly, patients with HPV-positive tumors tend to have better outcomes than those with HPV-negative ones, but the immune-related reasons for this difference remain unclear. In this study, we performed a focused reanalysis of publicly available single-cell RNA sequencing data, complemented by original protein-level validation, to examine natural killer (NK) cells in head and neck tumors. We discovered that HPV-positive tumors harbor a specialized NK cell population that supports patient survival, whereas HPV-negative tumors produce signals that block NK cell function. These findings help explain why the two cancer types behave differently and point toward new immune-based treatments tailored to a patient’s HPV status.

## 1. Introduction

HNSCC ranks as the 5th most common cancer worldwide by incidence (both sexes), arising from the mucosal epithelium of the oral cavity, pharynx, and larynx [1,2]. While historically linked to tobacco and alcohol, HPV infection is now an established etiological factor that defines a distinct clinical entity [3,4,5]. A striking clinical paradox in HNSCC is that HPV-positive (HPV^+^) tumors, despite their viral origin, are associated with a more favorable prognosis and higher survival rates compared to HPV-negative (HPV^−^) tumors. Growing evidence suggests that this dichotomy may arise from fundamental differences in the composition and functional state of the tumor immune microenvironment (TIME), which is profoundly influenced by HPV status. For example, in cervical and oropharyngeal cancers, HPV has been shown to foster a more immunologically active TIME, characterized by enhanced lymphocyte infiltration and antigen presentation [6,7]. However, the precise biological mechanisms underlying this prognostic disparity remain poorly understood, representing a critical knowledge gap that impedes the development of targeted therapeutic strategies [5,8,9].

The TIME serves as a crucial battlefield in HNSCC progression. Among its diverse cellular components, NK cells have emerged as a key regulatory population whose functional activity may be differentially modulated by HPV status. As essential effectors of the innate immune system, NK cells provide a first line of defense through major histocompatibility complex (MHC)-independent recognition and elimination of malignant cells [10,11,12]. Their anti-tumor potential is further enhanced through crosstalk with other immune cells, including T cells and dendritic cells [13,14,15]. Despite this capability, NK cell function is often compromised within the solid tumor microenvironment. Tumors can evade NK cell surveillance through various mechanisms, such as impaired recruitment due to genetic alterations, such as TP53 loss [16,17], and the secretion of inhibitory factors that dampen cytotoxic activity [18,19]. A fundamental unresolved question is whether the functional state of NK cells is differentially regulated by HPV status—and if so, whether this could help explain the prognostic paradox observed in HNSCC.

To address this question, we performed an NK-cell-centric re-analysis of publicly available high-resolution single-cell RNA sequencing (scRNA-seq) data to construct a comprehensive atlas of the tumor-infiltrating NK (TINK) cell landscape in HPV^+^ and HPV^−^ HNSCC—a focused analysis distinct from previous broad immune landscape studies of HNSCC in published GEO datasets (GSE139324, GSE164690) [20,21]. This targeted approach allowed us to identify prognostically relevant NK cell subsets within a complex, dynamic ecosystem where the spatial and temporal arrangement of cells is as important as their identity. Ultimately, this study aims to provide a mechanistic foundation for the observed prognostic differences in HNSCC, and our findings identify four core novel contributions: (1) the first high-resolution NK cell atlas for HPV-stratified HNSCC; (2) a protective CX3CR1^+^KLRB1^dim^ NK subset in HPV^+^ HNSCC; (3) the CLEC2–KLRB1 axis as a novel targetable immune evasion mechanism in HPV^−^ HNSCC; (4) original immunohistochemistry (IHC) validation of this axis in an independent institutional cohort. These insights lay a mechanistic foundation for precision immunotherapies tailored to HPV status in HNSCC [22,23]. This is particularly relevant as the field of NK cell immunotherapy rapidly advances, with CAR-NK cells targeting EGFR and CD44v6 showing potent anti-tumor activity [24,25], and novel engineering strategies incorporating chemokine receptors to improve tumor infiltration [26,27]. Furthermore, combination therapies pairing NK cells with checkpoint inhibitors or monoclonal antibodies, such as cetuximab, are under active clinical investigation to overcome the multifaceted immunosuppression of HNSCC [28].

## 2. Materials and Methods

### 2.1. Patient Characteristics

This study included two independent patient cohorts with confirmed HPV status, distinguished by experimental purpose (computational re-analysis vs. original IHC validation).

#### 2.1.1. Discovery Cohort (Cohort A)

A total of 28 HNSCC patient samples (18 HPV^−^, 10 HPV^+^) were assembled from two public Gene Expression Omnibus (GEO) scRNA-seq datasets (GSE139324 [20], GSE164690 [21]) for computational re-analysis of the NK cell landscape. Clinical characteristics (including anatomical subsite, age, and gender) of this cohort are summarized in Supplementary Table S1 (Revised). HPV status for the discovery cohort was determined by the original clinical annotations of the GEO datasets (p16 IHC and/or RNA-seq-based viral transcript detection).

#### 2.1.2. Validation Cohort (Cohort B)

Formalin-fixed, paraffin-embedded (FFPE) HNSCC tissue sections were retrieved from 10 HNSCC patients (5 HPV^−^, 5 HPV^+^) undergoing surgical resection at the Department of Head and Neck Surgery, Cancer Hospital of the Chinese Academy of Medical Sciences (Shenzhen Hospital) for original IHC validation of the CLEC2–KLRB1 axis. Full clinical characteristics of this cohort (anatomical subsite, TNM stage, HPV testing method) are provided in Supplementary Table S1. HPV status for the validation cohort was confirmed by testing p16 IHC in the institutional clinical laboratory.

The study protocol adhered to the principles of the Declaration of Helsinki and was formally approved by the Ethics Committee of the Third People’s Hospital of Shenzhen (Approval No. 2021-055). Written informed consent was obtained from all participants in the validation cohort prior to sample collection.

### 2.2. Data Collection

Single-cell RNA sequencing (scRNA-seq) data were obtained from two publicly accessible Gene Expression Omnibus (GEO) datasets: GSE139324 [20] and GSE164690 [21]. Based on clinical annotations from the original publications, a discovery cohort of 28 HNSCC patient samples with confirmed HPV status was assembled for analysis, including 18 HPV-negative and 10 HPV-positive samples. Detailed sample identifiers, GEO accession numbers, and HPV status are provided in Supplementary Table S1.

For cross-cohort consistency, HPV status was harmonized across all datasets used in this study: (1) Discovery Cohort (GEO): original clinical annotations (p16 IHC) [20,21]; (2) Validation Cohort (institutional): p16 IHC; (3) TCGA-HNSC cohort: integrated p16 IHC/HPV DNA status from TIMER 2.0. Detailed clinical annotations for all cohorts are provided in Supplementary Table S1.

### 2.3. Quality Control and RNA-Seq Data Processing

#### 2.3.1. scRNA-seq Data Preprocessing and Quality Control

Raw gene expression matrices were downloaded from GEO and processed using the Seurat package (v3.0) in R (v4.2.3). Low-quality cells with >20% mitochondrial reads or <200 detected genes were excluded. After initial filtering, the dataset consisted of 69,872 cells expressing 48,513 genes. To enrich for immune cells, a subsequent filter was applied to retain only CD45^+^ cells, resulting in a final dataset of 64,513 cells. Mitochondrial, ribosomal, and hemoglobin-related genes were removed before further analysis.

#### 2.3.2. Data Integration and Batch Correction

To mitigate batch effects, data integration was performed using Seurat (v4). Individual samples were normalized and scaled separately, and the top 2000 variable features were identified. Integration anchors were determined using the FindIntegrationAnchors function, and a batch-corrected expression matrix was generated with the IntegrateData function.

#### 2.3.3. Dimensionality Reduction, Clustering, and Annotation

The integrated data were scaled, and principal component analysis (PCA) was conducted. The top 28 principal components were selected for downstream analysis. Clustering was performed using the Louvain algorithm with a resolution of 0.6, yielding 23 distinct clusters. Visualization was performed via Uniform Manifold Approximation and Projection (UMAP). Clusters were annotated based on canonical marker genes from the CellMarker database [29].

#### 2.3.4. Re-Analysis of T and NK Cell Subsets

T and NK cells were subsetted from the integrated data for further high-resolution analysis. This subset underwent independent re-clustering following the same pipeline, using 30 principal components and a resolution of 0.6. A total of 22 refined clusters were obtained and annotated as specific T or NK cell subtypes based on marker gene expression and functional enrichment analysis of differentially expressed genes (DEGs).

### 2.4. Functional Enrichment Analysis

Gene Ontology (GO) and pathway enrichment analyses were conducted using the DAVID bioinformatics platform (v2023q4) on cluster-specific DEGs. Enriched terms in the Biological Process, Cellular Component, and Molecular Function categories were considered significant at a Benjamini–Hochberg-adjusted *p*-value < 0.05.

### 2.5. Public Bulk RNA-seq Data Analysis (TIMER, TCIA)

#### 2.5.1. Survival Analysis

Associations between immune infiltration and patient survival were evaluated using the Survival module of TIMER 2.0. Analyses were conducted separately for HPV-stratified TCGA-HNSC cohorts.

Multivariable Cox proportional hazards models were constructed to validate the prognostic robustness of NK cell signatures, with adjustment for key clinical covariates: age, tumor TNM stage, and smoking status. All survival analyses were performed separately for HPV^+^ and HPV^−^ subgroups of the TCGA-HNSC cohort, and results of univariable and multivariable models are presented in Supplementary Figure S3 (Revised).

#### 2.5.2. Immune Infiltration and Gene Correlation Analysis

In the TCGA-HNSC cohort, HPV status was defined using integrated p16 IHC/HPV DNA status from TIMER 2.0, with cases with ambiguous or missing annotations excluded from HPV-stratified analyses. Immune cell proportions were estimated using TIMER’s Estimation module and the CIBERSORT algorithm via The Cancer Immunome Atlas (TCIA), and correlations between gene expression and immune cell abundance were assessed using the Gene module in TIMER.

### 2.6. Pseudotime Estimation

Developmental trajectories of NK cells were inferred using the Monocle2 R package (v2.18.0) [30], with analyses performed separately for HPV^−^ and HPV^+^ samples to avoid cross-group bias. Key technical parameters for trajectory inference were defined as follows: (1) root cell selection: CD56^bright^ NK subset (the most immature NK subset, identified by highest expression of immaturity markers including CD56 and IL2RB); (2) trajectory alignment: trajectories were inferred independently for HPV^+^ and HPV^−^ groups and then visually compared for conserved/divergent differentiation paths; (3) robustness checks: developmental ordering was confirmed to be stable across the top 10–30 principal components and alternative root cell selections. Trajectories were constructed via reverse graph embedding to order cells by transcriptional similarity.

### 2.7. Cell–Cell Interaction Analysis

Cell–cell communication between NK subsets and tumor cells was inferred based on the expression of 7807 curated ligand–receptor pairs from the CellChat database [31]. The interaction score calculation was defined as the product of the average ligand expression in tumor cells and the average receptor expression in NK subsets. Significant interactions were identified using a one-sample Wilcoxon signed-rank test (FDR < 0.05, interaction score > 0.5), with patient-level heterogeneity control: only interactions significant across multiple (≥3) patient samples were retained for downstream analysis. A null model (1000 permutation tests) was constructed to validate the statistical significance of identified ligand–receptor pairs [19,32], and it was used as a positive control to confirm the validity of the analysis pipeline.

### 2.8. Immunohistochemistry (IHC) Staining

IHC was performed on formalin-fixed, paraffin-embedded HNSCC tissue sections. Following deparaffinization, rehydration, and antigen retrieval, sections were incubated overnight at 4 °C with primary antibodies against CD69 (Abcam, ab233396, 1:250) and CLEC2D (Abcam, Cambridge, UK, ab239222, 1:250). HRP-conjugated secondary antibodies were applied, and slides were scanned using the Vectra Automated Quantitative Pathology System (PerkinElmer, Shelton, CT, USA). Staining was performed on samples from the independent Validation Cohort (Cohort B, *n* = 10), with CLEC2C and CLEC2D as the primary targets for validating the CLEC2–KLRB1 axis (CD69 as a positive control for immune cell infiltration). Quantification of staining intensity was performed using ImageJ software (v1.54f).

### 2.9. Statistical Analysis

Statistical analyses were performed in SPSS (v19.0) and R (v4.2.3). Group comparisons used Student’s *t*-test or chi-square test as appropriate. Correlations were assessed using Pearson or Spearman tests. A *p*-value < 0.05 was considered statistically significant, with *p* < 0.05 (*), *p* < 0.01 (**), *p* < 0.001 (***), and *p* < 0.0001 (****); all survival analyses were adjusted for multiple comparisons using the Benjamini–Hochberg method.

## 3. Results

### 3.1. The Immune Landscape of HNSCC Reveals Altered NK Cell Infiltration in HPV^+^ Tumors

**Figure 1:**
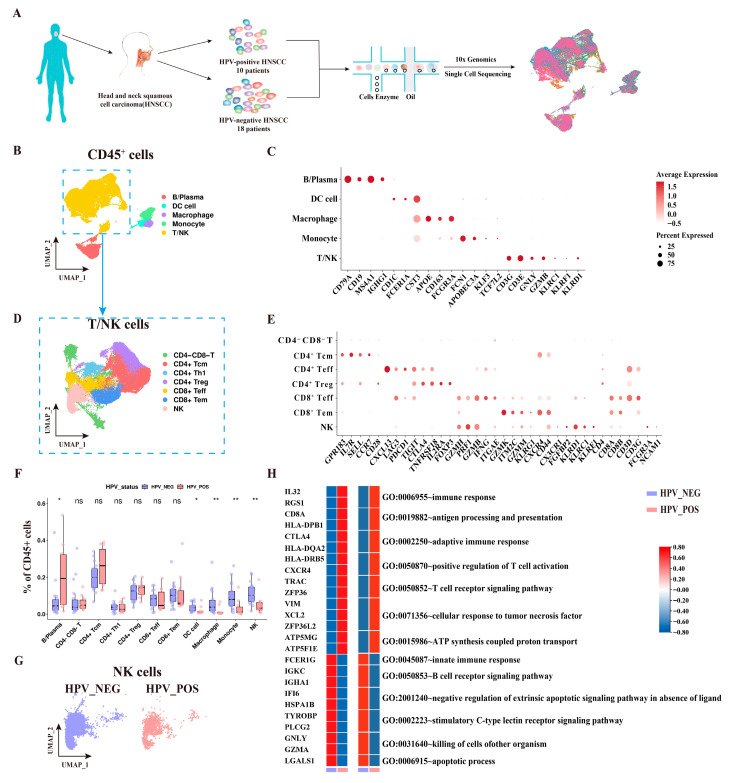
Single-cell immune landscape of the Discovery Cohort (Cohort A, n = 28, GEO scRNA-seq data). A total of 64,513 single cells were recovered from 28 samples. (**A**) Tumor-infiltrating CD45+ cells were sorted from single-cell suspensions prepared from tissue samples and subjected to single-cell RNA-seq. (**B**) Uniform Manifold Approximation and Projection (UMAP) plot displaying the major cell types. Each dot represents an individual cell, and the colors indicate different cell populations. DC, dendritic cells; NK, natural killer cells. (**C**) The dot plot shows the distribution of characteristic genes for B/Plasma, DC cell, Macrophage, Monocyte, and T/NK cells. (**D**) UMAP plot illustrating the detailed cell types in T and NK cells. Tcm, central memory T cells; Treg, regulatory T cells; Teff, effector T cells; Tem, effector memory T cells. (**E**) The dot plot shows the distribution of characteristic genes for T and NK cells. (**F**) Boxplot demonstrating the frequency of immune cells in the CD45+ cell populations using HPV^−^ and HPV^+^ HNSCC scRNA datasets. The statistical significance is indicated in the figure: ns (not significant) when *p* > 0.05, * when *p* <= 0.05, and ** when *p* <= 0.01. (**G**) UMAP plot displaying NK cells in HPV^−^ and HPV^+^ HNSCC. Each dot represents an individual cell, and the colors indicate different HPV infection statuses. (**H**) Heatmap showing differentially expressed genes and functional enrichment in NK cells from HPV^−^ and HPV^+^ HNSCC.

We analyzed single-cell RNA sequencing (scRNA-seq) data from 28 HNSCC patients, including 18 HPV^−^ and 10 HPV^+^ cases, to characterize the tumor microenvironment immune landscape. After quality control, 64,513 CD45^+^ cells were retained for downstream analysis (Figure 1A). Unsupervised clustering identified five major immune lineages: B/plasma cells, dendritic cells (DCs), macrophages, monocytes, and a combined T/NK cell population (Figure 1B,C). The T/NK compartment constituted over 70% of all CD45^+^ cells across samples (Figure S1A). Further subclustering of this population resolved distinct T and NK cell subsets, including CD4^−^CD8^−^ T cells, CD4^+^ central memory (Tcm), effector (Teff), and regulatory (Treg) cells, as well as CD8^+^ Teff, memory (Tem), and NK cells (Figure 1D,E).

A comparative analysis of immune cell proportions between HPV^−^ and HPV^+^ HNSCC revealed several notable differences. The fraction of B/plasma cells was significantly elevated in HPV^+^ tumors, consistent with prior reports linking B cell infiltration to a favorable prognosis in this subgroup [33]. In contrast, proportions of dendritic cells, macrophages, monocytes, and NK cells were significantly lower in HPV^+^ samples (Figure 1F,G). Notably, a distinct subset of macrophages expressing TCR and CD3 has been previously identified in HPV^+^ HNSCC, potentially participating in phagocytosis and regulating TCR signaling [34]. No significant differences in the frequencies of T cell subsets were observed between the two groups.

Given the observed reduction in NK cell infiltration in HPV^+^ tumors, we next examined NK cell function. Functional enrichment analysis of DEGs in NK cells from HPV^+^ versus HPV^−^ HNSCC revealed that genes upregulated in HPV^+^ NK cells were enriched for pathways related to antigen presentation, adaptive immune response, and T cell activation. Conversely, genes upregulated in HPV^−^ NK cells were associated with innate immunity and antiviral responses (Figure 1H).

### 3.2. Single-Cell Analysis Identifies Four Functionally Distinct NK Cell Subsets in HNSCC

**Figure 2:**
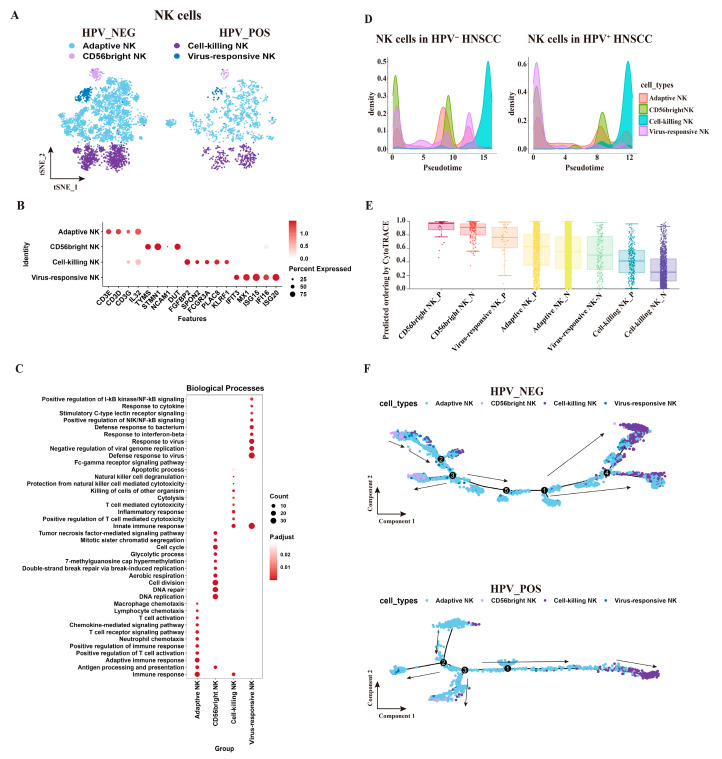
Characterization of Single-Cell Expression Profiles of NK Subsets in HPV^−^ and HPV^+^ HNSCC. A total of 7932 single cells were recovered from 28 samples. (**A**) The t-distributed Stochastic Neighbor Embedding (t-SNE) plot displays four NK subsets in HPV^−^ and HPV^+^ HNSCC. Each dot represents an individual cell, and the colors indicate different cell populations. (**B**) Dot plot showing the functional enrichment of NK subsets. (**C**) Dot plot illustrating the distribution of characteristic genes for NK subsets. (**D**) Pseudotime analysis of NK subsets in HPV^−^ (**left**) and HPV^+^ HNSCC (**right**). (**E**) Predicted ordering by CytoTRACE, which orders NK cells based on their developmental potential from most mature (lowest values) to most immature (highest values). Boxplots show the median and interquartile range, and whiskers extend from the minimum to the maximum. (**F**) Simulated differentiation trajectory analysis of NK subsets in HPV^−^ (**up**) and HPV^+^ HNSCC (**down**).

To further resolve NK cell heterogeneity, 7932 NK cells were re-clustered, yielding four transcriptionally distinct subsets: adaptive NK, cell-killing NK, CD56^bright^ NK, and virus-responsive NK (Figure 2A). This deep characterization aligns with 2024 scRNA-seq findings demonstrating significant transcriptomic differences between putative circulating NK cells and TINKs, the latter of which often co-express multiple inhibitory receptors and exhaustion-associated genes, such as TIM-3, upon entering the TME [35]. Additionally, our analysis of virus-responsive subsets complements recent studies on tissue-resident NK (trNK) cells in HPV-positive HNSCC, which exhibit unique functional properties for local immunosurveillance [36]. Functional annotation revealed specialized profiles: the cell-killing subset was enriched in cytotoxicity-related terms (e.g., cell lysis, apoptosis); the adaptive NK subset showed enrichment for adaptive immunity pathways; the virus-responsive subset was linked to C-type lectin receptor signaling and defense responses; and the CD56^bright^ subset was associated with cell-cycle processes (Figure 2B,C).

Pseudotime trajectory analysis indicated similar overall differentiation paths between HPV^−^ and HPV^+^ NK cells (Figure 2D). In both groups, CD56^bright^ NK cells localized to the trajectory origin, consistent with a less-differentiated state, whereas cell-killing NK cells occupied the terminus, reflecting terminal differentiation (Figure 2F). Developmental potential analysis using CytoTRACE further indicated that the virus-responsive NK subset in HPV^−^ tumors exhibited lower differentiation status than its HPV^+^ counterpart (Figure 2E).

### 3.3. A Circulating CX3CR1-Expressing NK Cell Subset Is Associated with Favorable Prognosis in HPV^+^ HNSCC

**Figure 3:**
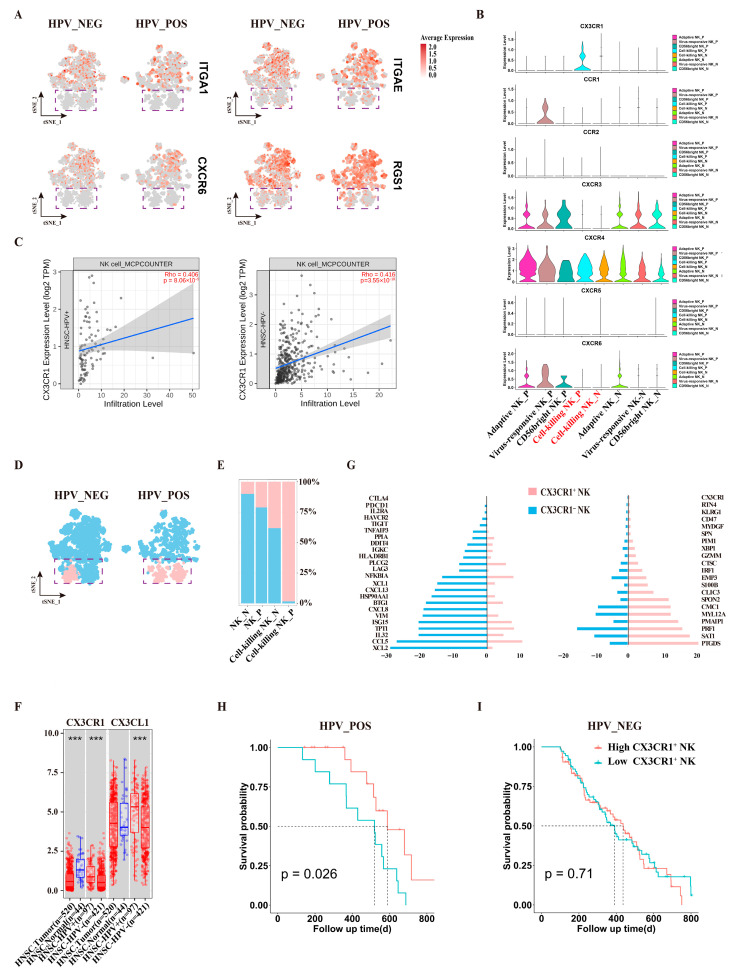
Heterogeneity of TINK subsets in HPV^−^ and HPV^+^ HNSCC. (**A**) T-SNE plots illustrate ITGA1, CXCR6, ITGAE, and RGS1 expression levels in NK subsets of HPV^−^ and HPV^+^ HNSCC. The purple outlines the dashed line of the cell-killing NK cells in the figure. (**B**) Violin plot illustrating the expression of CX3CR1, CCR1, CCR2, CXCR3, CXCR4, CXCR5, and CXCR6 in different subpopulations of NK cells. (**C**) Scatter plot demonstrating the impact of the expression level of CX3CR1 and CX3CL1 on the infiltration of NK cells in HPV^−^ and HPV^+^ HNSCC. (**D**,**E**) T-SNE plot and histogram show the proportion of CX3CR1^+^ NK subsets in HPV^−^ and HPV^+^ HNSCC. (**F**) The bar chart shows the expression of CX3CR1 and CX3CL1 in HPV^−^ and HPV^+^ HNSCC and normal tissues (*p* < 0.001 (***)). (**G**) The gene expression profiling of CX3CR1^+^ and CX3CR1^−^ NK Subtypes. (**H**,**I**) Kaplan–Meier plots illustrate the relationship between the high and low infiltration of CX3CR1^+^ NK cells and prognosis.

To distinguish tissue-resident from circulating NK cells, we evaluated expression of tissue-resident markers (ITGA1, CXCR6, ITGAE, RGS1). The cell-killing NK subset showed the lowest expression of these markers, suggesting a putative circulating-like phenotype (Figure 3A). Among chemokine receptors, CX3CR1 was highly expressed in cell-killing NK cells, particularly in HPV^+^ tumors (Figure 3B). Consistent with a role in NK cell trafficking, both CX3CR1 and its ligand CX3CL1 correlated positively with NK cell infiltration scores in bulk transcriptomic data (Figure 3C).

We subsequently subdivided the cell-killing NK population into CX3CR1^+^ and CX3CR1^−^ subsets (Figure 3D). The proportion of CX3CR1^+^ cells within this subset was markedly higher in HPV^+^ HNSCC (~95%) compared to HPV^−^ tumors (Figure 3E). Bulk-tissue analysis confirmed elevated expression of both CX3CR1 and CX3CL1 in HPV^+^ HNSCC (Figure 3F). The CX3CR1^+^ subset exhibited higher expression of cytotoxicity-related genes (PRF1, GZMM), whereas the CX3CR1^−^ subset showed upregulation of inhibitory/exhaustion markers (TIGIT, PDCD1, CTLA4) (Figure 3G).

Clinically, high infiltration of CX3CR1^+^ cell-killing NK cells was associated with significantly improved overall survival in HPV^+^ HNSCC patients. No such correlation was observed in the HPV^−^ cohort (Figure 3H,I).

### 3.4. IHC Validation of the CLEC2–KLRB1 Axis in an Independent HNSCC Validation Cohort

**Figure 4:**
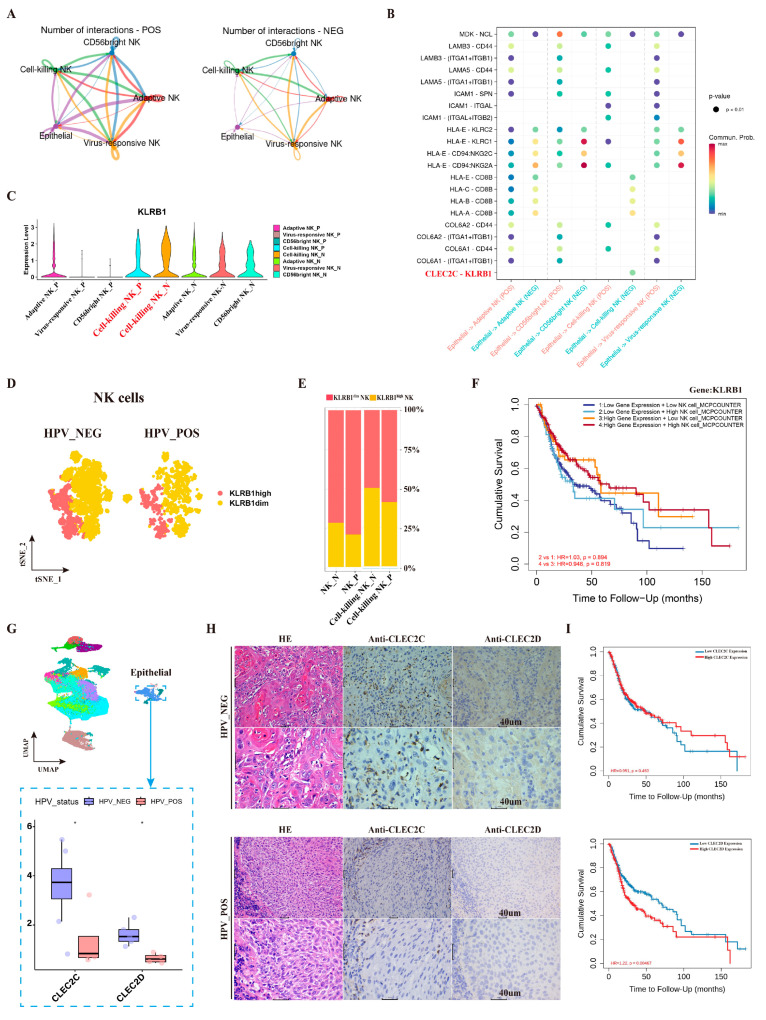
Tumor-immune cellular interaction between NK subsets and tumor cells in the HPV^−^ and HPV^+^ HNSCC tumor microenvironment. (**A**) Circle plot displaying the interactions between NK cell subsets and tumor cells in HPV^−^ and HPV^+^ HNSCC. (**B**) Bubble plots show ligand–receptor pairs between NK subsets and tumor cells in HPV^−^ and HPV^+^ HNSCC. The color indicates the mean expression of ligand and receptor genes, and the dot size represents the statistical significance of interactive molecular pairs. (**C**) The violin plot shows the expression levels of KLRB1 across different NK cell subpopulations. (**D**,**E**) T-SNE plot and histogram show the proportion of CX3CR1^+^ NK subsets in HPV^−^ and HPV^+^ HNSCC. (**F**) Kaplan–Meier plots demonstrate the relationships among KLRB1 expression, NK cell infiltration levels, and prognosis. (**G**) Bubble plots of CLEC2C and CLEC2D expression levels in HPV^−^ and HPV^+^ HNSCC cells. (**H**) IHC staining results from the Validation Cohort (Cohort B, n = 10, institutional samples) depict the expression patterns of CLEC2C and CLEC2D in HPV^−^ and HPV^+^ tumor cells. (**I**) Kaplan–Meier plots demonstrate the association between CLEC2C and CLEC2D expression and prognosis.

Cell–cell communication analysis revealed a reduced overall interaction frequency between NK cells and tumor cells in HPV^−^ tumors compared with HPV^+^ tumors (Figure 4A). In HPV^−^ HNSCC, we observed significant enrichment of the inhibitory HLA-E: CD94-NKG2A/C axis [19] (Figure 4B). Further analysis highlighted specific engagement of the CLEC2B/C/D-KLRB1 inhibitory axis between tumor cells and cell-killing NK cells, predominantly in HPV^−^ disease (Figure S4).

While KLRB1 expression on NK cells did not differ between HPV^−^ and HPV^+^ tumors (Figure 4C–E) and showed no prognostic association (Figure 4F), expression of the ligands CLEC2C and CLEC2D was significantly upregulated on tumor cells in the HPV^−^ cohort (Figure 4G). Immunohistochemistry (IHC) confirmed higher protein levels of CLEC2C and CLEC2D in HPV^−^ tissues (Figure 4H). Analysis of TCGA-HNSC data further indicated that high CLEC2D expression correlated with poor prognosis, whereas CLEC2C showed no significant association (Figure 4I).

### 3.5. Prognostically Favorable CX3CR1^+^KLRB1^dim^ NK Cells Are Enriched in HPV^+^ HNSCC

**Figure 5:**
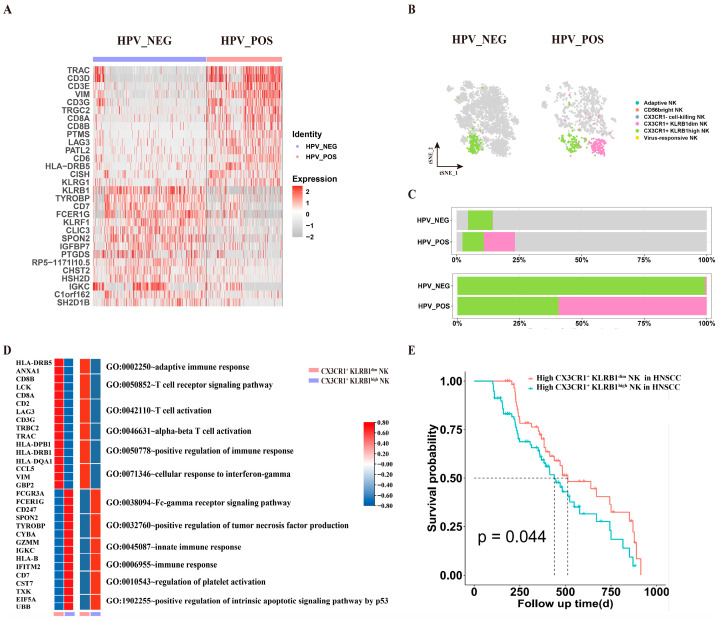
Differential Functional Profiling of CX3CR1^+^ and CX3CR1^−^ NK Cell Subtypes in HPV^−^ and HPV^+^ HNSCC. (**A**) Heatmap showing the gene Expression Profiling of CX3CR1^+^ NK Subtypes in HPV^−^ and HPV^+^ HNSCC. (**B**,**C**) T-SNE plot and histogram show the proportion of CX3CR1^+^ KLRB1high and CX3CR1^+^ KLRB1^dim^ NK subsets in HPV^−^ and HPV^+^ HNSCC. (**D**) Heatmap plot showing the differentially expressed genes and functional enrichment of CX3CR1^+^ NK Subtypes in HPV^−^ and HPV^+^ HNSCC. (**E**) Kaplan–Meier plots demonstrate the relationship between the infiltration of CX3CR1^+^ KLRB1high and CX3CR1^+^ KLRB1^dim^ NK subsets in HPV^−^ and HPV^+^ HNSCC and their prognostic significance.

We further stratified CX3CR1^+^ cell-killing NK cells based on KLRB1 expression. In HPV^+^ tumors, CX3CR1^+^ NK cells were predominantly KLRB1-low/negative (KLRB1^dim^), whereas HPV^−^ tumors harbored a higher frequency of CX3CR1^+^KLRB1^high^ NK cells (Figure 5A). Accordingly, CX3CR1^+^ NK cells were classified into CX3CR1^+^KLRB1^dim^ and CX3CR1^+^KLRB1^high^ subsets (Figure 5B). The CX3CR1^+^KLRB1^dim^ subset was significantly more abundant in HPV^+^ HNSCC (Figure 5C).

Functional enrichment analysis showed that the CX3CR1^+^KLRB1^dim^ subset was associated with adaptive immunity and T cell activation, whereas the CX3CR1^+^KLRB1^high^ subset was linked to TNF production and platelet activation (Figure 5D). Clinically, higher infiltration of CX3CR1^+^KLRB1^dim^ NK cells correlated with improved patient survival, while the CX3CR1^+^KLRB1^high^ subset showed no prognostic association (Figure 5E).

### 3.6. HPV-Dependent Modulation of NK–Tumor Cell Interactions

**Figure 6:**
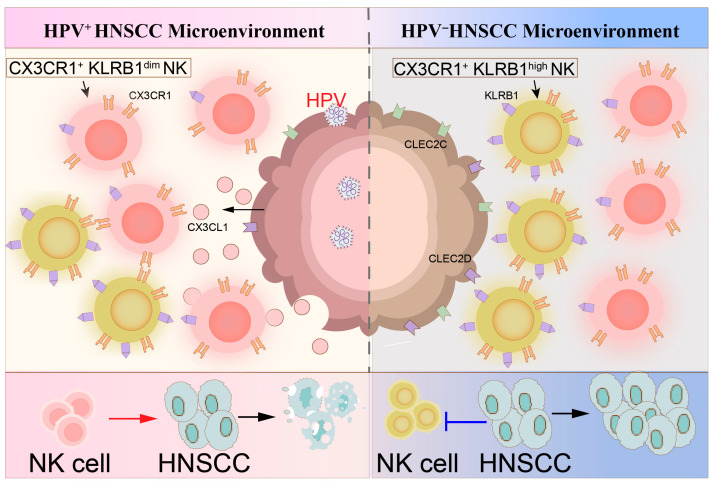
A schematic representation depicting the interaction between NK cells and HPV^−^ and HPV^+^ HNSCC cells.

We integrated the above findings into a schematic model summarizing the HPV-dependent interplay between NK cells and tumor cells (Figure 6). In HPV^+^ HNSCC, tumor cells exhibit downregulated CLEC2B/C/D ligands and upregulated CX3CL1. NK cells, especially the cell-killing subset, express high levels of CX3CR1. The resulting CX3CR1^+^KLRB1^dim^ NK cells, which exhibit a putative circulating-like transcriptional signature, are inferred to be recruited via the CX3CL1-CX3CR1 axis, exhibit enhanced cytotoxicity, and are associated with a favorable prognosis. In contrast, HPV^−^ tumors upregulate surface CLEC2B/C/D ligands, which engage KLRB1 on NK cells, suppressing cytotoxic function and promoting an exhausted phenotype, thereby contributing to immune evasion and poorer clinical outcomes.

## 4. Discussion

This study presents a high-resolution atlas of the NK cell landscape in HNSCC, revealing a pronounced functional dichotomy governed by tumor HPV status. By dissecting NK cell heterogeneity into four functionally specialized subsets—adaptive, cell-killing, CD56^bright^, and virus-responsive—we provide a mechanistic perspective on the well-established prognostic differences between HPV^+^ and HPV^−^ disease. In the prognostically favorable HPV^+^ tumors, we observed a significant enrichment of a protective CX3CR1^+^KLRB1^dim^ cytotoxic NK cell population. The predominance of this subset implies a robust, putative circulating-like anti-tumor NK response. Moreover, the prominence of an “adaptive” NK subset in HPV^+^ HNSCC, characterized by gene expression signatures linked to T-cell activation and antigen presentation, further supports the notion of a highly coordinated and effective immune microenvironment. Although the expression of T-cell-associated genes such as CD3D and CD3E by NK cells is unconventional, similar phenotypes have been described in memory-like NK cells that arise during chronic viral infections, suggesting a unique maturation state shaped by the HPV^+^ tumor milieu [37,38].

In sharp contrast, the tumor microenvironment in HPV^−^ HNSCC appears to actively subvert NK cell function. Although the cell-killing NK subset is proportionally present in HPV^−^ tumors, its cytotoxic efficacy is likely impaired by a specific and potent immune-evasion mechanism. Our analysis identified upregulation of the CLEC2 ligands CLEC2C and CLEC2D on HPV^−^ tumor cells, which engage the inhibitory receptor KLRB1 on NK cells. This interaction, computationally predicted and validated by immunohistochemistry, highlights a dominant, targetable axis of NK cell suppression specific to this more aggressive disease subtype [32,39]. Beyond tumor-mediated inhibition, emerging evidence from 2025 suggests that family members, such as CLEC2B, expressed by cancer-associated fibroblasts, may contribute to an immunosuppressive niche by inhibiting NK cells via the KLRB1 receptor [40]. Consequently, blockade of the CLEC2–KLRB1 interaction represents a rational strategy to rejuvenate innate immunity, potentially dismantling stromal barriers that form physical obstacles to immune infiltration [41,42,43]. This mechanism likely synergizes with other established immunosuppressive components of the tumor microenvironment, such as regulatory T cells and tumor-associated macrophages [44,45], collectively fostering a multi-layered resistance to NK-mediated tumor elimination.

The observed reduction in NK cell infiltration within HPV^+^ tumors appears to contrast with previous reports of elevated circulating NK cell numbers in the peripheral blood of the same patient population. This discrepancy may reflect a fundamental distinction between systemic immunity and local intra-tumoral immune activity. One plausible explanation is that chronic immune activation in the HPV^+^ microenvironment—potentially driven by factors such as CXCL10—induces NK cell death upon tumor entry, leading to lower steady-state intra-tumoral counts despite a larger systemic reservoir [41,46,47]. This highlights a key limitation of the present study: the absence of paired peripheral blood samples. Future investigations comparing circulating and tumor-infiltrating NK cells from the same individuals will be essential to fully elucidate NK cell trafficking, activation, and functional fate in HNSCC.

In summary, this work delineates the HPV-dependent heterogeneity of the NK cell compartment in HNSCC. It provides mechanistic insights into the divergent clinical outcomes observed between HPV^+^ and HPV^−^ patients. Our findings can be distilled into two central, testable hypotheses for future research: (1) the CX3CR1^+^KLRB1^dim^ cytotoxic NK subset is associated with enhanced anti-tumor immunity in HPV^+^ HNSCC and serves as a candidate prognostic biomarker pending prospective validation; (2) the CLEC2C/D–KLRB1 axis is associated with NK cell suppression in HPV^−^ HNSCC and represents a candidate therapeutic target, subject to functional in vitro/in vivo confirmation.

Despite the mechanistic insights provided by our high-resolution NK cell atlas of HPV-stratified HNSCC, several limitations of this study warrant consideration and temper the interpretation of our findings. First, there is near-complete collinearity between HPV status and anatomical subsite in our Discovery Cohort: 9/10 HPV^+^ cases are oropharyngeal (base of tongue, tonsil, or oropharynx), whereas all 18 HPV^−^ cases are non-oropharyngeal (predominantly oral cavity: tongue, floor of mouth, buccal mucosa, oral cavity)—a reflection of the clinical epidemiology of HNSCC. While we performed subsite-stratified sub-analyses where possible, observed differences in NK cell transcriptional signatures and functional subsets may partially reflect tissue-specific mucosal immune contextures rather than HPV biology alone. Second, our study focuses on intra-tumoral NK cells and lacks paired peripheral blood mononuclear cells (PBMCs) from the same patients, which limits our ability to track systemic-to-local trafficking of NK cell subsets (e.g., the CX3CR1^+^ population) and fully map their functional fate in the TIME. Third, the sample size of our independent IHC Validation Cohort (n = 10) is relatively small, and while this cohort provides critical protein-level confirmation of the CLEC2–KLRB1 axis, it is underpowered for stratification by anatomical subsite or tumor stage. Finally, our Discovery Cohort relies on published scRNA-seq datasets with pre-defined clinical annotations, which limits our ability to adjust for additional covariates (e.g., treatment history, smoking intensity) that may influence NK cell biology in HNSCC. Furthermore, the HPV^+^ cases in our Validation Cohort (Cohort B) are all of laryngeal origin (3 larynx, 1 epiglottis, 1 vocal cord)—an atypical subsite distribution for HPV-positive HNSCC, which is epidemiologically dominated by oropharyngeal disease. This limits the extent to which the IHC findings in Cohort B can be considered a direct validation of the oropharyngeal HPV^+^ NK cell phenotypes identified in Cohort A.

Future studies addressing these limitations are essential to validate our findings and advance their clinical translation. Specifically, large-scale, prospectively designed, subsite-matched cohorts of HPV^+^ and HPV^−^ HNSCC are needed to disentangle HPV-specific effects from site-specific effects on NK cell signatures. Integrating paired PBMC and tumor tissue samples will enable characterization of NK cell trafficking between the systemic and local immune compartments, and functional in vitro/in vivo studies of the CLEC2–KLRB1 axis will further validate its role as a targetable immune evasion mechanism in HPV^−^ HNSCC. Additionally, multi-omics analyses (e.g., scATAC-seq, proteomics) will provide a more comprehensive understanding of the epigenetic and post-transcriptional regulation of NK cell subsets in HPV-stratified HNSCC.

## 5. Conclusions

In conclusion, our study systematically delineates the heterogeneous intra-tumoral NK cell landscape in HNSCC and identifies an HPV-associated functional dichotomy in NK cell biology that correlates with divergent clinical outcomes. Via NK-cell-centric re-analysis of scRNA-seq data and original IHC validation in an independent cohort, we identified four transcriptionally distinct NK cell subsets and two key HPV-stratified immune phenotypes: (1) a cytotoxic CX3CR1^+^KLRB1^dim^ NK subset is specifically enriched in HPV^+^ HNSCC and serves as a protective prognostic biomarker (with independent prognostic value confirmed by multivariable Cox analysis); (2) HPV^−^ HNSCC tumors upregulate CLEC2C/CLEC2D ligands to engage the inhibitory KLRB1 receptor on NK cells, forming a novel targetable immune evasion axis validated at the protein level by IHC.

These findings provide a mechanistic framework for understanding the prognostic disparity between HPV^+^ and HPV^−^ HNSCC and highlight translational opportunities for precision immunotherapy. However, clinical translation of these findings requires validation in large, subsite-matched prospective cohorts to disentangle HPV-specific effects from anatomical site-dependent immune contextures. The CX3CR1^+^KLRB1^dim^ NK subset is a promising prognostic indicator for HPV^+^ HNSCC, and the CLEC2–KLRB1 axis represents a novel therapeutic target for HPV^−^ HNSCC—with functional in vitro/in vivo studies needed to confirm its therapeutic potential. Collectively, our work underscores the critical importance of HPV stratification in dissecting tumor–NK cell crosstalk in HNSCC and lays the groundwork for future personalized immune-based strategies tailored to the distinct immunological landscapes of HPV^+^ and HPV^−^ disease.

## Data Availability

The single-cell RNA sequencing datasets analyzed in this study are publicly available in the NCBI Gene Expression Omnibus (GEO) under accession numbers GSE139324 and GSE164690. The clinical and immunohistochemistry data supporting the findings of this study are available from the corresponding author upon reasonable request, subject to ethical and privacy restrictions.

## Supplementary Materials

The following supporting information can be downloaded at: https://www.mdpi.com/article/10.3390/cancers18050845/s1, Table S1: Clinical Characteristics of the Study Participants; Figure S1: Proportion of immune cells across patients; Figure S2: Proportion of NK cell subsets in HPV^+/−^ HNSCC; Figure S3: Cellular interaction between NK subsets and other cell clusters in the HPV^+/−^ HNSCC tumor microenvironment; Figure S4: Overview of the CLEC2B/C/D-KLRB1 ligand–receptor pairs between cell-killing NK subsets and other cell types in the HPV^+/−^ head and neck tumor microenvironment.

## Abbreviations

The following abbreviations are used in this manuscript:

HPV	Human Papillomavirus
NK	Natural Killer
trNK	tissue-resident NK
TINK	tumor-infiltrating NK
HNSCC	Head and Neck Squamous Cell Carcinoma
TIME	Tumor Immune Microenvironment
MHC	Major Histocompatibility Complex
scRNA-seq	Single-cell RNA Sequencing
GEO	Gene Expression Omnibus
DEG	Differentially Expressed Genes
