# Supplementary material for: Distinct NK Cell Signatures Define Prognosis in HPV-Positive Versus HPV-Negative Head and Neck Cancer
Source: Cancers (Basel). 2026 Mar 5;18(5):845. doi: 10.3390/cancers18050845 (PMC12984458; doi:10.3390/cancers18050845)
Supplement: Supplementary file 1 [file cancers-18-00845-s001.zip › cancers-4148957-supplementary.pdf]

cancers

MDPI

Table S1. Clinical Characteristics of the Study Participants.

HN #	Gender	Age	Disease site	Pathologic Stage	Pathological Node	HPV (p16 IHC)
			**Cohort A**			
1	Male	45	Oral cavity	T1	N2b	Neg
2	Male	50	Floor of the mouth	T4	N0	Neg
3	Female	66	Floor of the mouth	T4	N0	Neg
4	Male	48	Buccal mucosa	T4	N1	Neg
5	Male	45	Tongue	T3	N2b	Neg
6	Male	43	Larynx	T4	N2c	Neg
7	Male	74	Lower gum	T4	N3b	Neg
8	Female	60	Tongue	T3	N2a	Neg
9	Male	80	Oral cavity	TX	NX	Neg
10	Male	56	Tongue	T3	N2B	Neg
11	Female	57	Tongue	T3	N3B	Neg
12	Male	35	Tongue	T3	N0	Neg
13	Male	62	Supraglottis	T3	N0	Neg
14	Female	77	Mandible	T4A	N2C	Neg
15	Female	75	Tongue	T3	N0	Neg
16	Male	55	Tongue	T3	N0	Neg
17	Male	80	Buccal mucosa	T2	N0	Neg
18	Female	69	Floor of the mouth	T2	N1	Neg
19	Male	68	Base of tongue	T2	N2b	Pos
20	Male	62	Base of tongue	T1	N2b	Pos
21	Male	49	Base of tongue	T1	N2a	Pos
22	Male	66	Tonsil	T2	N1	Pos
23	Male	61	Tongue	T4	N1	Pos
24	Male	52	Base of tongue	T1	N1	Pos
25	Male	75	Tonsil	T2	N0	Pos
26	Male	59	Tonsil	T1	N1	Pos
27	Male	42	Oropharynx	T2	N1	Pos
28	Male	52	Oropharynx	T1	N1	Pos
			**Cohort B**			
1	Male	58	Larynx	T2	N0	Neg
2	Male	54	Right pyriform sinus	T3	N0	Neg

3	Male	75	Larynx	T4	N0	Neg
4	Female	53	Lower gingival cancer	T3	N1	Neg
5	Female	63	Larynx	T3	N0	Neg
6	Female	49	Larynx	T2	N0	Pos
7	Female	66	Epiglottis	T2	N0	Pos
8	Male	67	Larynx	T3	N0	Pos
9	Male	66	Left vocal cord	T3	N0	Pos
10	Male	63	Larynx	T1	N0	Pos

https://doi.org/10.3390/cancers18050845

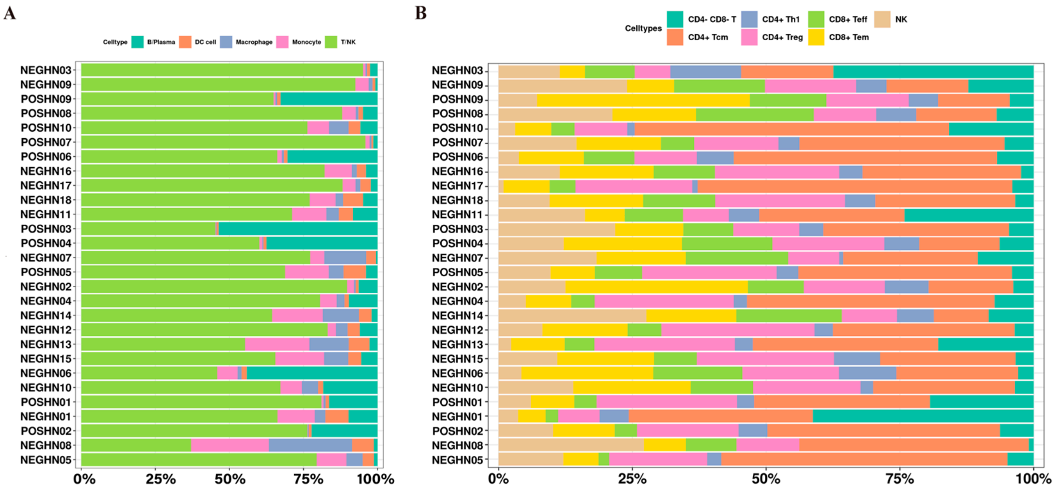

Figure S1. Proportion of immune cells across patients. (**A**) Bar plots of the proportion of cell type by patient. (**B**) Similar to (A), for NK cell and T cell subsets.

https://doi.org/10.3390/cancers18050845

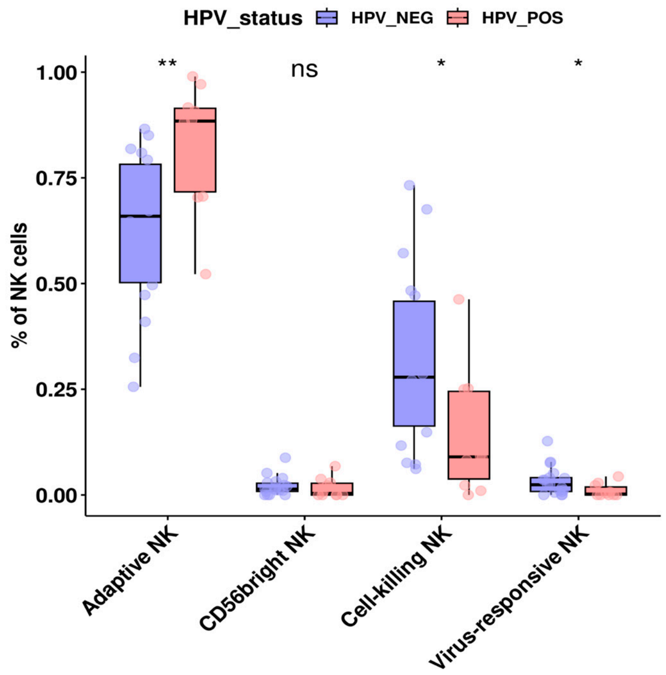

Figure S2. Proportion of NK cell subsets in HPV⁻ and HPV⁺ HNSCC. ns: $p > 0.05$, *: $p <= 0.05$, **: $p <= 0.01$.

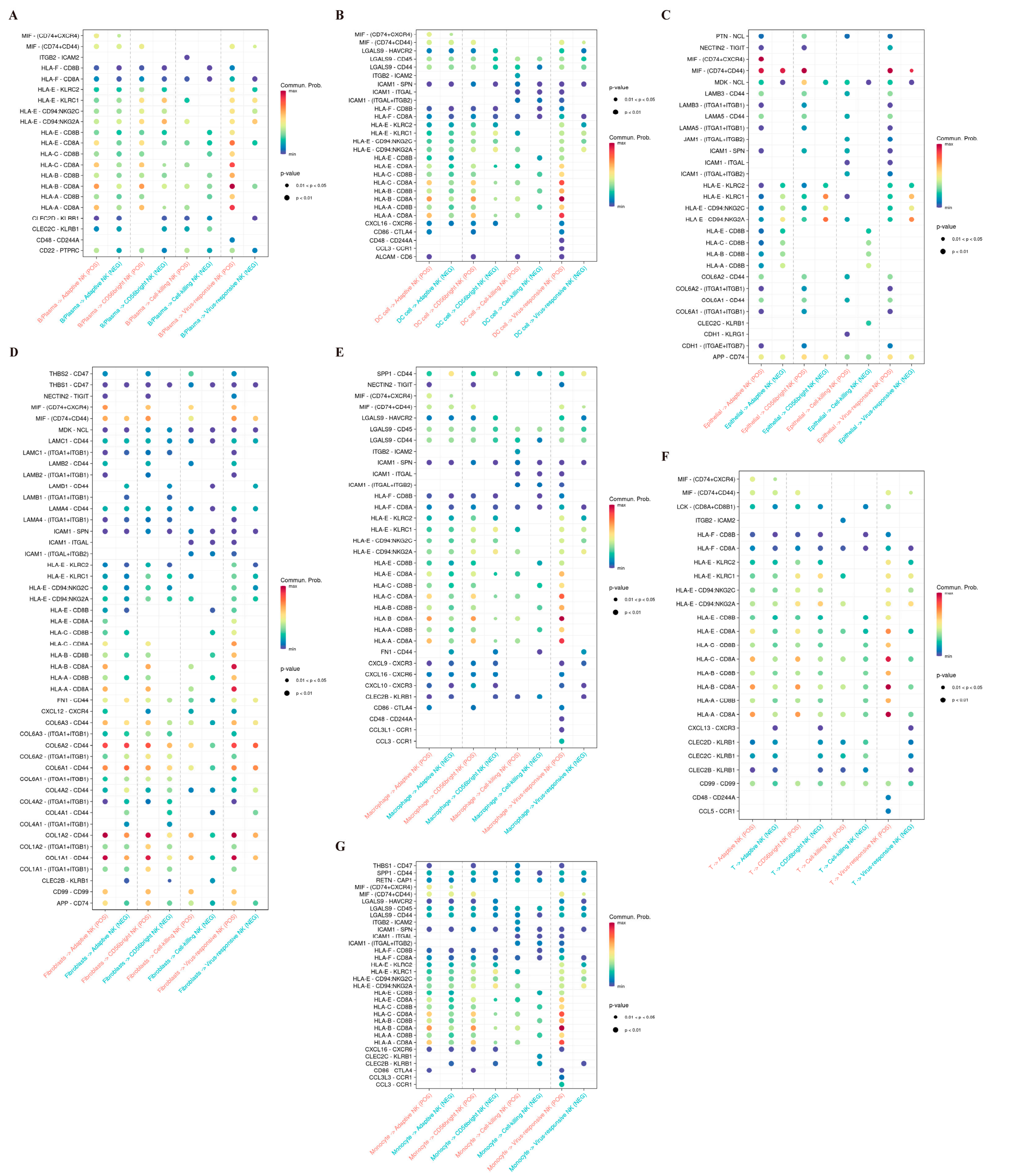

Figure S3. Cellular interaction between NK subsets and other cell clusters in the HPV^{-} and HPV^{+} HNSCC tumor microenvironment. (**A**) Bubble plots show ligand-receptor pairs between NK subsets and B/plasma cells. (**B**) Bubble plots show ligand-receptor pairs between NK subsets and DC cells. (**C**) Bubble plots show ligand-receptor pairs between NK subsets and Epithelial cells. (**D**) Bubble plots show ligand-receptor pairs between NK subsets and Fibroblasts. (**E**) Bubble plots show ligand-receptor pairs between NK subsets and Macrophage cells. (**F**) Bubble plots show ligand-receptor pairs between NK subsets and T cells. (**G**) Bubble plots show ligand-receptor pairs between NK subsets and Monocyte cells.

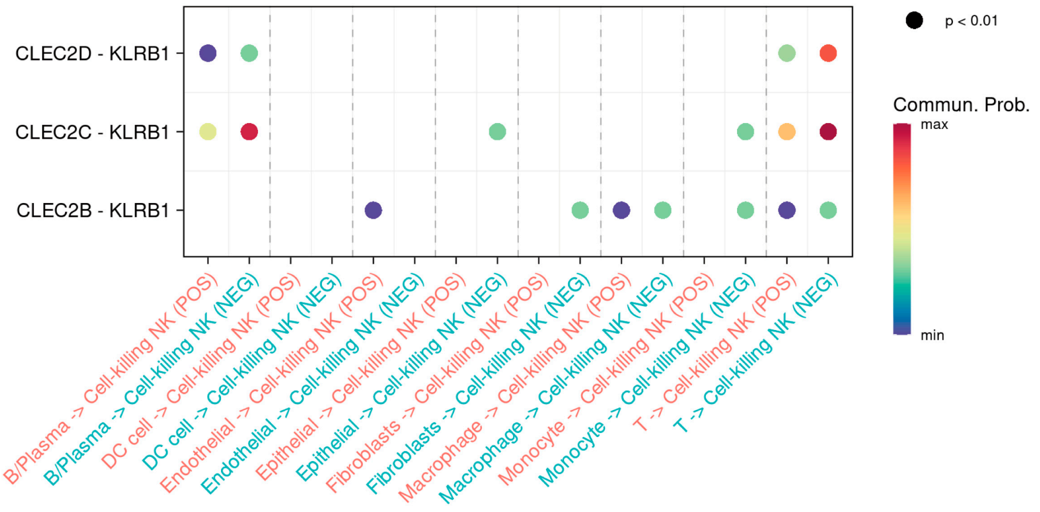

Figure S4. Overview of the CLEC2B/C/D-KLRB1 ligand-receptor pairs between cell-killing NK subsets and other cell types in the HPV^{-} and HPV^{+} head and neck tumor microenvironment. The color indicates the mean expression of ligand and receptor genes, and the dot size represents the statistical significance of interactive molecular pairs.

Disclaimer/Publisher's Note: The statements, opinions and data contained in all publications are solely those of the individual author(s) and contributor(s) and not of MDPI and/or the editor(s). MDPI and/or the editor(s) disclaim responsibility for any injury to people or property resulting from any ideas, methods, instructions or products referred to in the content.
